# Ceramide structure dictates glycosphingolipid nanodomain assembly and function

**DOI:** 10.1038/s41467-021-23961-9

**Published:** 2021-06-16

**Authors:** Senthil Arumugam, Stefanie Schmieder, Weria Pezeshkian, Ulrike Becken, Christian Wunder, Dan Chinnapen, John Hjort Ipsen, Anne K. Kenworthy, Wayne Lencer, Satyajit Mayor, Ludger Johannes

**Affiliations:** 1 Institut Curie, PSL Research University, U1143 INSERM, UMR3666 CNRS, Cellular and Chemical Biology unit, Paris, Cedex France; 2 National Centre for Biological Sciences (NCBS), Bangalore, India; 3 Monash Biomedicine Discovery Institute, Faculty of Medicine, Nursing and Health Sciences, Monash University, Clayton/Melbourne, VIC Australia; 4 European Molecular Biological Laboratory Australia (EMBL Australia), Monash University, Clayton/ Melbourne, VIC Australia; 5 Division of Gastroenterology, Boston Children’s Hospital, Boston, MA USA; 6 Groningen Biomolecular Sciences and Biotechnology Institute and Zernike Institute for Advanced Materials, University of Groningen, Groningen, Netherlands; 7 MEMPHYS/PhyLife, Department of Physics, Chemistry and Pharmacy, University of Southern Denmark, Odense M, Denmark; 8 Center for Membrane and Cell Physiology, University of Virginia, Charlottesville, VA USA; 9 Harvard Medical School, Boston, MA USA; 10 Harvard Digestive Diseases Center, Boston, MA USA

**Keywords:** Membrane structure and assembly, Super-resolution microscopy

## Abstract

Gangliosides in the outer leaflet of the plasma membrane of eukaryotic cells are essential for many cellular functions and pathogenic interactions. How gangliosides are dynamically organized and how they respond to ligand binding is poorly understood. Using fluorescence anisotropy imaging of synthetic, fluorescently labeled GM1 gangliosides incorporated into the plasma membrane of living cells, we found that GM1 with a fully saturated C16:0 acyl chain, but not with unsaturated C16:1 acyl chain, is actively clustered into nanodomains, which depends on membrane cholesterol, phosphatidylserine and actin. The binding of cholera toxin B-subunit (CTxB) leads to enlarged membrane domains for both C16:0 and C16:1, owing to binding of multiple GM1 under a toxin, and clustering of CTxB. The structure of the ceramide acyl chain still affects these domains, as co-clustering with the glycosylphosphatidylinositol (GPI)-anchored protein CD59 occurs only when GM1 contains the fully saturated C16:0 acyl chain, and not C16:1. Thus, different ceramide species of GM1 gangliosides dictate their assembly into nanodomains and affect nanodomain structure and function, which likely underlies many endogenous cellular processes.

## Abstract (web summary)

Gangliosides such as GM1 present in the outer leaflet of the plasma membrane of eukaryotic cells are essential for many cellular functions and pathogenic interactions. Here the authors show that the acyl chain structure of GM1 determines the establishment of nanodomains when actively clustered by actin, which depended on membrane cholesterol and phosphatidylserine or superimposed by the GM1-binding bacterial cholera toxin.

## Introduction

The plasma membrane of eukaryotic cells is composed of a large number of lipid species that are asymmetrically distributed between the inner and outer leaflets^1^ as well as phase segregated laterally into functional domains^2–4^. These domains have been thought of as phase segregated regions of the membrane with a liquid-ordered (lo) character, that serve to sort specific components for a functional purpose^5^, and termed colloquially as ‘rafts’^5^. Several passive and active (ATP dependent) mechanisms have been proposed to explain how raft domains are built^4^. The dynamic cortical actin cytoskeleton compartmentalizes the plasma membrane by affecting diffusion^6,7^, and by influencing phase separation passively^8^ and actively^9^. It has also been proposed that the membrane composition is at near miscibility critical points exhibiting critical fluctuations^10^, and that interactions with cytoskeleton may act to preserve heterogeneity over a larger range of temperature and compositions^11,12^. The acto-myosin cortex renders an additional layer of active organization that can drive the formation of nanoclusters, as exemplified by studies on GPI-anchored proteins^9,13^. These nanoclusters assemble in a cholesterol-dependent manner and are coupled across the bilayer to the acto-myosin cortex by the abundant inner leaflet lipid phosphatidylserine^14^. Whether such acto-myosin contractility-based clustering mechanism applies more generally to other categories of lipids is unknown.

Glycosphingolipids (GSLs) are composed of complex oligosaccharide headgroups linked to ceramide^15^. Among their many functions, GSLs form the basis for the assembly of raft domains induced by the interaction with extracellular GSL-binding ligands. The best-studied examples for this are the bacterial Shiga and cholera toxins, which via their homopentameric B-subunits (STxB and CTxB, respectively) recognize the sugar parts of the GSLs globotriaosylceramide (Gb3) or monosialotetrahexosylganglioside (GM1), respectively, as their cellular receptors^16,17^. It has been recognized early on that CTxB, by binding to GM1, directly influences the phase behavior of multicomponent membrane systems^18,19^. STxB, CTxB, and the pentameric fold capsid protein VP1 of simian virus 40, have then been shown to induce GSL reorganization, membrane bending, and the formation of narrow tubular endocytic pits from which endocytic carriers originated in a clathrin-independent manner^20,21^. The same mechanism—termed glycolipid-lectin (GL-Lect) hypothesis^22^—also appears to operate for the cellular GSL-binding protein galectin-3, which drives the clathrin-independent endocytic uptake of cargo proteins such as CD44 and α5β1 integrin^23^.

How STxB and CTxB induce membrane bending has been studied in depth using all-atom molecular dynamics (aaMD) simulations, which provided in silico evidence for toxin-driven domain formation, i.e., nanoscale membrane patches enriched for Gb3 or GM1, respectively^24,25^. The oligomeric nature of STxB and CTxB with, respectively, 15 Gb3 or 5 GM1-binding sites per homo-pentamer, is critical for this nanoscale GSL clustering. GSL-bound toxin molecules then may cluster themselves via membrane-mediated physical processes including fluctuation forces^26^, entropic considerations^27^, and potentially also by direct protein–protein interaction^28^.

aaMD studies also pointed to a specific geometry of GSL-binding sites on STxB and CTxB homopentamers that imprints an element of negative inward-oriented curvature onto the membrane patch^24,25^. The attenuated toxicity in cholera toxins with reduced numbers of binding sites hints towards the functional significance of the CTxB-induced GM1 arrangement^29^. Grazing incidence X-ray diffraction measurements showed that STxB binding to a Gb3-containing monolayer reduces the area per lipid, which in a bilayer would be expected to lead to the generation of negative membrane curvature^28^. Both effects are expected to contribute to toxin-driven GSL-dependent membrane bending.

Unlike the case of proteins, the tools available to study the distribution of GSLs in their native environment of cellular membranes remain relatively limited. Methods such as freeze-fracture electron microscopy^30–32^ and high resolution imaging mass spectrometry^33^ can provide nano to micron-scale information about organization of GSLs, but are limited to fixed cells. To study the organization and dynamics of GSLs in living cells, a variety of fluorescent analogs have been developed^34,35^. However, lipids are notoriously sensitive to fluorescent labeling. For example, depending on their chemical identity and position, fluorescent tags can disrupt the association of GM1 with raft domains as well as its ability to be recognized by CTxB^36^.

In recent years, synthetic fluorescent headgroup-labeled GSLs have been developed that more faithfully model the behavior of their natural counterparts, such as sorting correctly into ordered membrane domains^29,35^. Studies with these types of headgroup-labeled lipids have revealed that the structure of the fatty acyl chains in the ceramide domain of GM1 controls its sorting into retrograde trafficking pathways^29^. They have also enabled detailed studies of the dynamics of GSLs at the plasma membrane and their interactions with GPI-anchored proteins at the single molecule level^34,37^. However, the importance of ceramide structure in dictating the dynamic nanoscale organization of GSLs within the plasma membrane, and how these change in response to toxin binding remains unexplored.

In the current study, we addressed these questions using GM1 and CTxB as a model. For this, we examined two fluorescent GM1 species that differ in ceramide structure, containing either C16:0 or C16:1 acyl chains, using fluorescence anisotropy-based homo-FRET measurements^38^. We found that in the absence of CTxB, GM1 C16:0, but not C16:1 formed nanoclusters that depended on membrane cholesterol and inner membrane leaflet phosphatidylserine, implicating their dependence on the cortical actin cytoskeleton. CTxB binding to GM1 induced the formation of higher order plasma membrane clusters for both GM1 species. In the case of GM1 C16:0, CTxB binding also led to co-clustering with the glycosylphosphatidylinositol (GPI)-anchored protein CD59. Remarkably, this was not observed when CTxB was bound to GM1 C16:1. Thus the structure of the acyl chain of the ceramide moiety in GSLs appears to play a decisive role in the nature of the lipid nanodomain assembly and therefore its function within the plane of the plasma membrane. We discuss the implications of these findings for the understanding of how the plasma membrane of eukaryotic cells may be functionally compartmentalized in relation to the generation of endocytic uptake carriers.

## Results

### Clustering of GM1 depends on the structure of its acyl chain

**Fig. 1:**
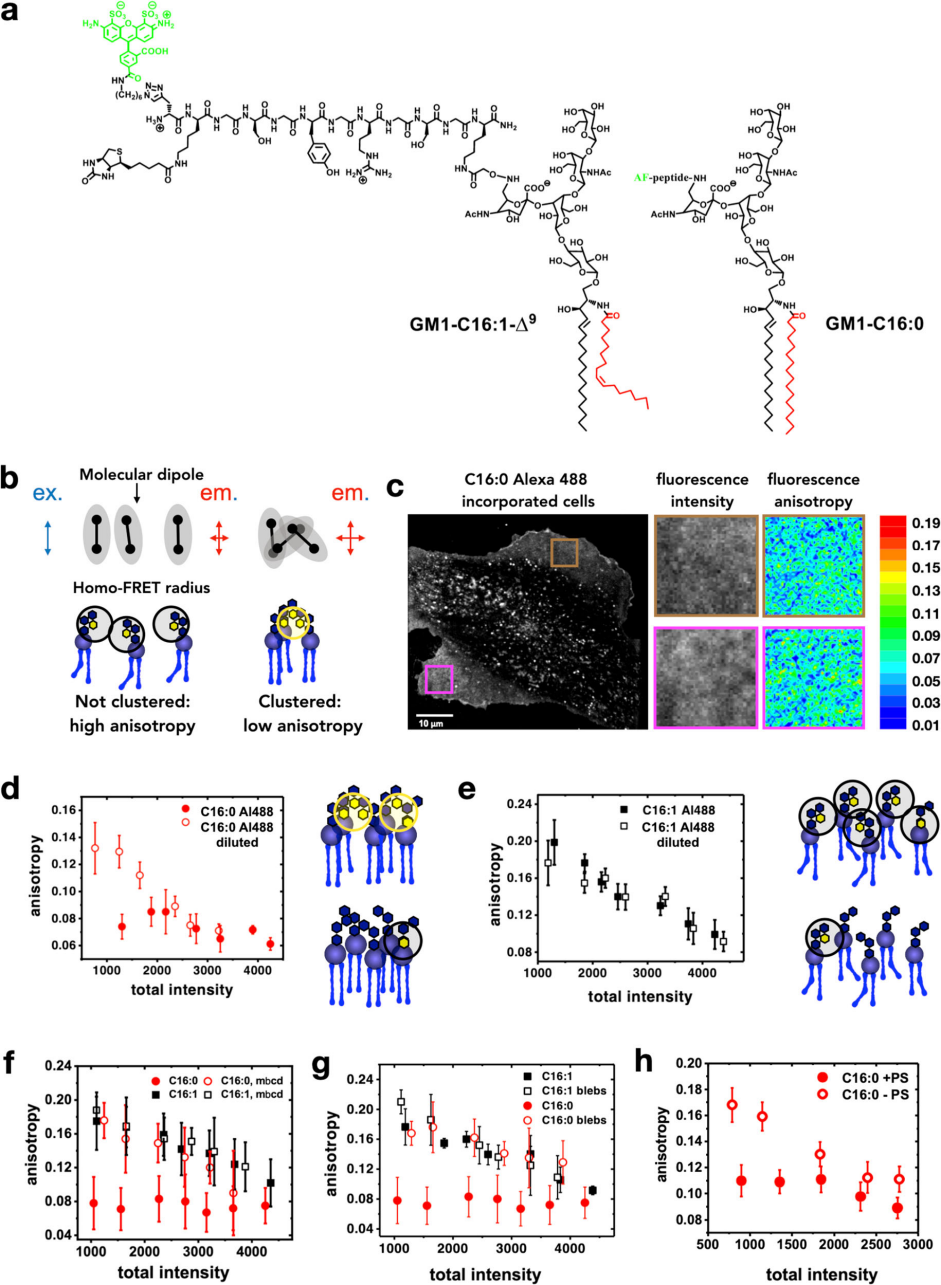
Measurement of clustering of fluorescent GM1 analogues in live cells. **a** Chemical structures of the GM1 analogues used in this study. **b** Schematic depicting loss of anisotropy upon clustering. The circle represents the radius within which homo-FRET can occur if two or more dyes are present within this circle. In the absence of clustering, the dyes are far apart, and emission is polarized. When clustered, multiple dye molecules are within the FRET distance, resulting in homo-FRET. Generally, this results in depolarization and therefore, lower anisotropy, unless clustering occurs in a manner that the orientations of the dye dipole are more rigidly locked, resulting in increased anisotropy. **c** A total emission intensity image of a cell incorporated with fluorescent C16: 0 GM1 with specific regions of interest (ROIs) for anisotropy mapping. **d** Scatter plot of anisotropy vs total intensity for 100% fluorescently labeled GM1 C16:0 (solid red circles), 80% unlabeled GM1 C16:0 with 20% fluorescently labeled Alexa488-GM1 C16:0 (open red circles). **e** Scatter plot of anisotropy vs total intensity for 100% fluorescently labeled GM1 C16:1 (solid black squares), 80% unlabeled GM1 C16:1 with 20% fluorescently labeled Alexa488-GM1 C16:1 (open black squares). **f** Scatter plots of anisotropy vs total intensity for Alexa488-GM1 C16:1 (black squares) and Alexa488-GM1 C16:0 (red circles) under no perturbation (solid symbols) or mβcd treatment conditions (open symbols). **g** Scatter plots of anisotropy vs total intensity for Alexa488-GM1 C16:1 (black squares) and Alexa488-GM1 C16:0 (red circles) of intact membranes (solid symbols) and blebs (open symbols). **h** Scatter plots of anisotropy vs total intensity for Alexa488-GM1 C16:0 in the presence (solid red circles) and absence of PS (open red circles). Absence of PS abrogates active Alexa488-GM1 C16:0 clustering. In **d**–**h**, means ± standard deviation is shown. *n* = 20 cells over 4 independent experiments for **d** and **e**, *n* = 15 cells over 6 independent experiments for **f**–**h**.

To study dynamic molecular level nano and meso-scale GSL organization at the plasma membrane, we incorporated fluorescently labeled GM1 species into living cells. The clustering behavior of two GM1 species differing in ceramide structure by a single *cis*-double bond in the acyl chain of the ceramide moiety (GM1 C16:0 and GM1 C16:1; Fig. 1a) could thereby be analyzed. Both species were labeled with Alexa488 fluorophore coupled via a peptide linker arm to the sialic acid residue of the oligosaccharide moiety^39^ (Fig. 1a). The synthetic GM1 species were added exogenously to a GSL-deficient cell line, GM95, using a protocol previously shown to result in functional incorporation of synthetic lipid analogs into the plasma membrane, as evidenced by reconstitution of cholera toxin function^29^, the expected phase behavior for the different GM1 species in giant plasma membrane vesicles (Supplementary Fig. 1), and the fact that CTxB bound to fluorescently labeled GM1 species with the same sensitivity as to the unlabeled counterparts (Supplementary Fig. 2). For the current study, we titrated the amounts of the Alexa488-GM1 lipids such as to obtain similar CTxB binding as on naturally GM1-positive mouse embryonic fibroblasts or HeLa cells (Supplementary Fig. 3).

Fluorescence emission anisotropy measurements were used to detect fluorescence resonance energy transfer (FRET) between fluorophores of neighboring molecules^38^ (Fig. 1b). In this type of measurement plane polarized light excites photo-selected fluorophores (Supplementary Fig. 4). A microscope that is capable of resolving fluorescence emission intensity in the parallel and perpendicular orientations to the excitation axis is then used to generate total fluorescence emission intensity (Fig. 1c, left) and anisotropy maps (Fig. 1c, right) of the membrane-incorporated fluorescently labeled GM1 species. The pseudo-colored fluorescence anisotropy images correspond to the boxed regions of interest in the total fluorescence emission image. Cool colors represent low fluorescence anisotropy, i.e., high GM1 clustering. Homo-FRET typically results in a loss of fluorescence anisotropy^40^, unless ordering of dye dipoles occurs along with clustering^41,42^. Therefore, by mapping the extent of depolarization of fluorescence emission we obtain the regions of the membrane where clusters of the labeled lipid species are enriched (Fig. 1c, boxed regions; see below).

In an initial set of experiments, we compared the organization of GM1 C16:0 and GM1 C16:1 species. Alexa488-GM1 C16:0 consistently showed low anisotropy values that did not vary with the amount of GM1 incorporated into the membrane, as measured by the intensity of the Alexa488-GM1 fluorescence signal (Fig. 1d, solid red circles). This pattern is a strong signature for active clustering, e.g., driven by cortical acto-myosin dynamics^43^. In contrast, Alexa488-GM1 C16:1 showed low anisotropy values when present at high membrane levels (high fluorescence intensity), and anisotropy progressively and linearly increased in cell membranes containing less Alexa488-GM1 C16:1 (less fluorescence intensity) (Fig. 1e, solid black squares). This concentration-dependent (i.e., intensity-dependent) behavior typifies randomly distributed molecules that are brought within FRET proximity as the result of their concentration within the membrane by random proximity action, an effect sometimes referred to as bystander FRET or random proximity FRET^44,45^.

Of note, when cells were reconstituted with a 1:5 mix of Alexa488-labeled and non-fluorophore-labeled GM1 C16:0 (Fig. 1d, open red circles) or GM1 C16:1 (Fig. 1e, open black squares), the anisotropy curves showed in both cases concentration-dependent behaviors. For GM1 C16:0, this result is consistent with the hypothesis that each active nanocluster must be limited in size/content to only a few molecules of this GSL species, as found for GPI-anchored proteins by anisotropy measurements^46^, and by electron microscopy for GM1 and GM3 gangliosides^30^. For GM1 C16:1, the results with the 1:5 mix reinforces our interpretation that homo-FRET between these GSL species predominantly arises from concentration-dependent FRET occurring between randomly distributed molecules.

### Active clustering of GM1 depends on cholesterol, phosphatidylserine, and interdigitation

Our results so far suggest that GM1 C16:0 can assemble into active nanoclusters but GM1 C16:1 cannot. We next investigated if such GM1 C16:0 nanoclusters depended on membrane cholesterol. We found that the concentration-independent (intensity-independent) low anisotropy signature of Alexa488-GM1 C16:0 (Fig. 1f, solid red circles) was lost in cells that were incubated with mβCD to extract cholesterol from the plasma membranes (Fig. 1f, open red circles). This result indicated that the lipid was not actively clustered any more. Alexa488-GM1 C16:1 anisotropy (Fig. 1f, solid black squares) remained intensity-dependent (not actively clustered) upon cholesterol extraction (Fig. 1f, open black squares), as expected.

To test if the acto-myosin cortex may be involved in active clustering of Alexa488-GM1 C16:0, we compared anisotropy signals on cells to those obtained on plasma membrane blebs (i.e., plasma membrane extrusions of micron sizes) prepared from the same cells. Such blebs are devoid of actin^13^ and lack clusters of outer leaflet GPI-anchored proteins^43^. While active clustering of Alexa488-GM1 C16:0 was again observed on control cells (Fig. 1g, solid red circles), it was lost on blebs (open red circles). In contrast, no difference between cells and blebs was measured for Alexa488-GM1 C16:1 (Fig. 1g, solid versus open black squares, respectively) as expected for GM1 species that do not assemble into active nanodomains.

Cytosolic acto-myosin machinery couples via phosphatidylserine (PS) to GPI-anchored proteins in the exoplasmic leaflet of the plasma membrane^14^. To test whether PS was also involved in the actin-dependent clustering of Alexa488-GM1 C16:0, we performed anisotropy measurements in PSA-3 cells, which are PS-deficient^47^. The concentration-independent anisotropy signal of Alexa488-GM1 C16:0 was abolished in PSA-3 cells and restored when the cells were grown in conditions that restored PS levels (Fig. 1h; solid red circles are from PS-sufficient cells, and open red circles from PS-deficient cells). Thus, the active clustering of Alexa488-GM1 C16:0 depends on membrane cholesterol, actin, and PS. PS may be operating here in *trans*-bilayer coupling of lipids to the acto-myosin cortex^14^.

Such *trans*-bilayer coupling is based on the interdigitation of acyl chains from lipids on either side of the bilayer and is likely to cause *trans-*bilayer coupling of aggregated lipids at one leaflet with corresponding lipids at the other leaflet^8^. To test the potential for this in the GM1 system, we performed aaMD simulations of asymmetric bilayers containing 4% of GM1 in the upper monolayer (for detailed information on monolayer compositions, see Supplementary Tables 1 and 2). In all the studied cases, saturated GM1 (sGM1) lipids interdigitated more into the opposite monolayer compare to unsaturated GM1 (uGM1) (Supplementary Fig. 5, Supplementary Tables 3 and 4). For instance, for DOPC bilayers containing 4% of GM1 in the upper monolayer, we found that while the sphingosine chains of sGM1 and uGM1 interdigitated equally (0.43 versus 0.45, respectively), the acyl chains for sGM1 interdigitated more deeply compared to uGM1 (0.47 versus 0.25, respectively). We do note that the forces between two grafted layers of polymer brushes are repulsive due to an entropic effect. However, for a system such as the membranes that we have modeled, both entropic and enthalpy effects (e.g., van der Waals attraction) exist, and interdigitation happens as observed here when the enthalpy of the system overcomes the repulsive entropy.

To investigate the effect of cholesterol, we performed aaMD of bilayers containing GM1, POPC, and sphingomyelin in the upper monolayer, and POPE and POPS in the lower monolayer in the presence or the absence of cholesterol (for detailed information on monolayer compositions, see Supplementary Table 2). Our simulation results suggest that although cholesterol generally reduced interdigitation for all the lipids in the simulation, lipid chains of opposing monolayers effectively interacted with each other. We also found that in the absence of cholesterol, GM1 coupled equally with both POPS and POPE, while in the presence of cholesterol, GM1-POPS *trans*-bilayer coupling became significantly larger than GM1-POPE *trans*-bilayer coupling (Supplementary Fig. 6). These results strongly suggest that cholesterol enhances GM1-POPS *trans*-bilayer coupling, thereby contributing to the active organization of lipids in the extracellular leaflet, in agreement with experimental results published elsewhere^48^.

Taken together, the dependence of clustering of sGM1 on cholesterol and PS and its absence in blebs along with the interdigitation observed in the simulations suggest that sGM1 is more susceptible than uGM1s to modulation through active processes involving the coupling to the acto-myosin cytoskeleton.

### CTxB reorganizes GM1 molecules

We next examined the effect of CTxB binding on the organization and behavior of the different GM1 species. The CTxB homo-pentamer contains five identical GM1-binding sites^49^. CTxB is well known to influence phase behavior in GM1-containing membranes^18^, and to drive the intracellular trafficking of GM1 in intact cells^29^.

We therefore predicted that CTxB binding could have multiple effects on GM1 nanodomain formation: ordering of bound GM1, clustering of multiple GM1s under the protein to induce nanodomain assembly, and clustering of adjacent CTxB/GM1 complexes for nanodomain growth.

**Fig. 2:**
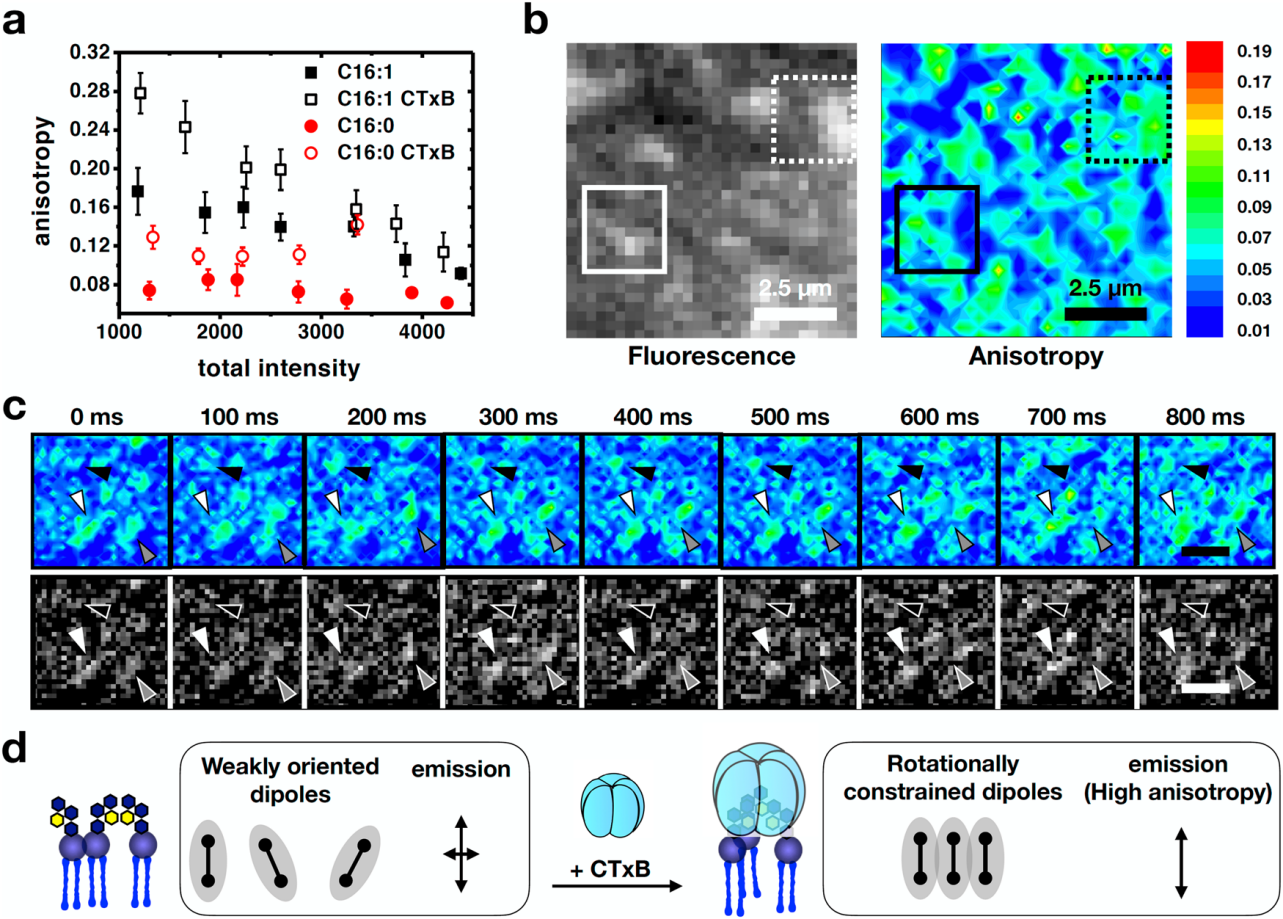
Effect of CTxB binding on GM1 clustering. **a** Scatter plot of anisotropy vs total intensity with (open symbols) or without CTxB (solid symbols) for Alexa488-GM1 C16:0 (red circles) and Alexa488-GM1 C16:1 (black squares). Note that upon CTxB binding, the anisotropy signal increases in all conditions. Means ± standard deviation is shown. *n* = 13 cells over 6 independent experiments. **b** Examples of total intensity and anisotropy images depicting a correlation between higher intensity and higher anisotropy upon CTxB binding referring to conditions of Fig. 2a. **c** Top: Anisotropic regions observed upon CTxB binding are highly stable in timescales of seconds and show dynamic rearrangements (arrowheads). Bottom: Corresponding intensity images that show higher intensities areas corresponding to areas of high anisotropy. Scale bars: 0.5 μm. **d** Schematic depicting gain of anisotropy upon CTXB-induced clustering that results in alignment of the molecular dipoles, and therefore increase in anisotropy, contrary the general decrease in anisotropy upon clustering.

The effect of CTxB on the organization of Alexa488-GM1 C16:0 and C16:1 was analyzed by anisotropy measurements on correspondingly reconstituted cells. In the absence of CTxB, Alexa488-GM1 C16:0 again showed concentration-independent anisotropy, indicative of the presence of active nanoclusters (Fig. 2a, solid red circles), whereas Alexa488-GM1 C16:1 exhibited concentration-dependent anisotropy consistent with homo-FRET between randomly distributed molecules (Fig. 2a, solid black squares). When bound to CTxB, the anisotropy signal for both GM1 species increased (Fig. 2a, open red circles for Alexa488-GM1 C16:0, and open black squares for Alexa488-GM1 C16:1). This increase in anisotropy in both cases was likely explained by a decrease in rotational diffusion of the Alexa488-GM1 headgroup induced by binding of the massive CTxB-subunit (see also below). Of note, clusters of Alexa488-GM1 C16:0 were apparent in total intensity images (Fig. 2b, black/white image). These clusters showed higher anisotropy values (Fig. 2b, pseudo-colored) and were stable over timescales of seconds (Fig. 2c, arrowheads), strongly suggesting that higher order clustering of GM1 indeed occurred as the result of CTxB binding (Fig. 2d).

**Fig. 3:**
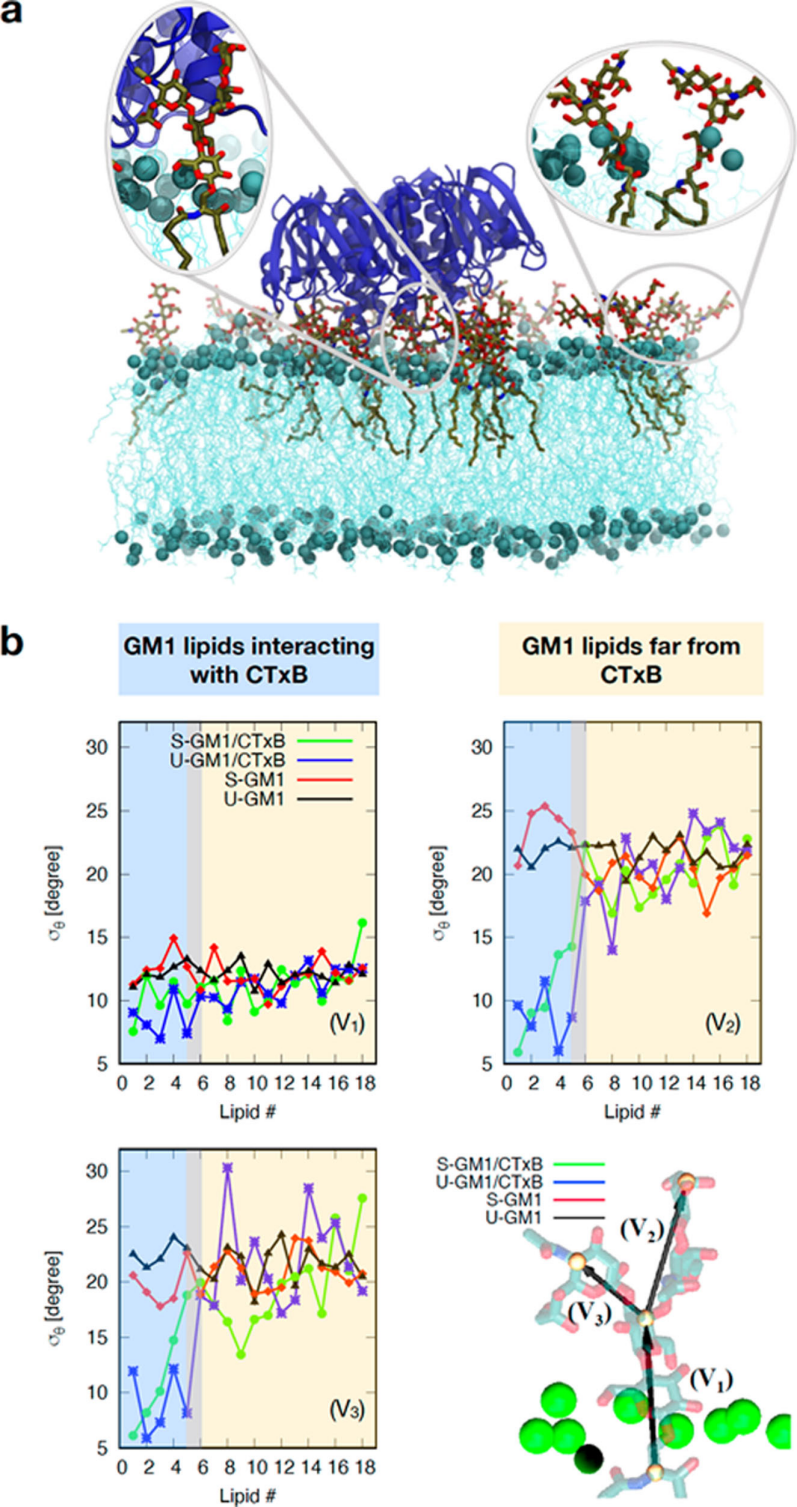
aaMD simulations of CTxB binding to GM1. **a** Snapshot of a CTxB bound to a lipid bilayer containing GM1. **b** The headgroup of GM1 by 3 vectors (V_1_, V_2_, V_3_). ${\sigma }_{\theta }$ is the standard deviation of the angle between these vectors and the bilayer normal vector. The graphs show that for lipids that are in contact with CTxB (lipids 1–5 in the green and blue curves), ${\sigma }_{\theta }$ becomes 2–4 time smaller. This indicates that CTxB restrains the fluctuations of the headgroups.

The potential for reducing the angular fluctuations of GM1 upon CTxB binding was further explored using aaMD simulations. The interaction of 1 CTxB molecule with 18 GM1 lipids (sGM1 or uGM1) in an asymmetric bilayer containing otherwise 430 1,2-dioleoylsn-glycero-3-phosphocholine (DOPC) lipids was simulated for 1 μsec (Fig. 3a). The 5 GM1-binding sites of CTxB were all saturated under these conditions. We then quantified the orientation of the GM1 headgroup relative to the bilayer normal in the presence and absence of CTxB (see graphics in Fig. 3b). Because not all GM1s are bound to CTxB, we also compared as an internal control the headgroup orientation for CTxB-bound GM1 (lipids 1–5 in Fig. 3b, shaded in blue) versus unbound GM1 (lipids 6–18, shaded in yellow). Distinct angular fluctuations (as demonstrated by low standard deviations) of CTxB-bound lipids were observed, when compared to the unbound lipids (Fig. 3b). This clearly indicated that CTxB restrained the fluctuations of the GM1 headgroups, which could explain the fluorescence anisotropy increase that was observed in our experiments in response to CTxB binding. Further, the simulation results showed that sGM1 area per lipid increased by 3.3 A^2^ due to the binding of CTxB, while for uGM1 this increase was only 0.7 A^2^ (Supplementary Table 5). Since the tendency was inverse for the anisotropy changes (Fig. 2a), we concluded that an area per lipid effect was unlikely to explain the observed higher anisotropy of the bound GM1 species.

**Fig. 4:**
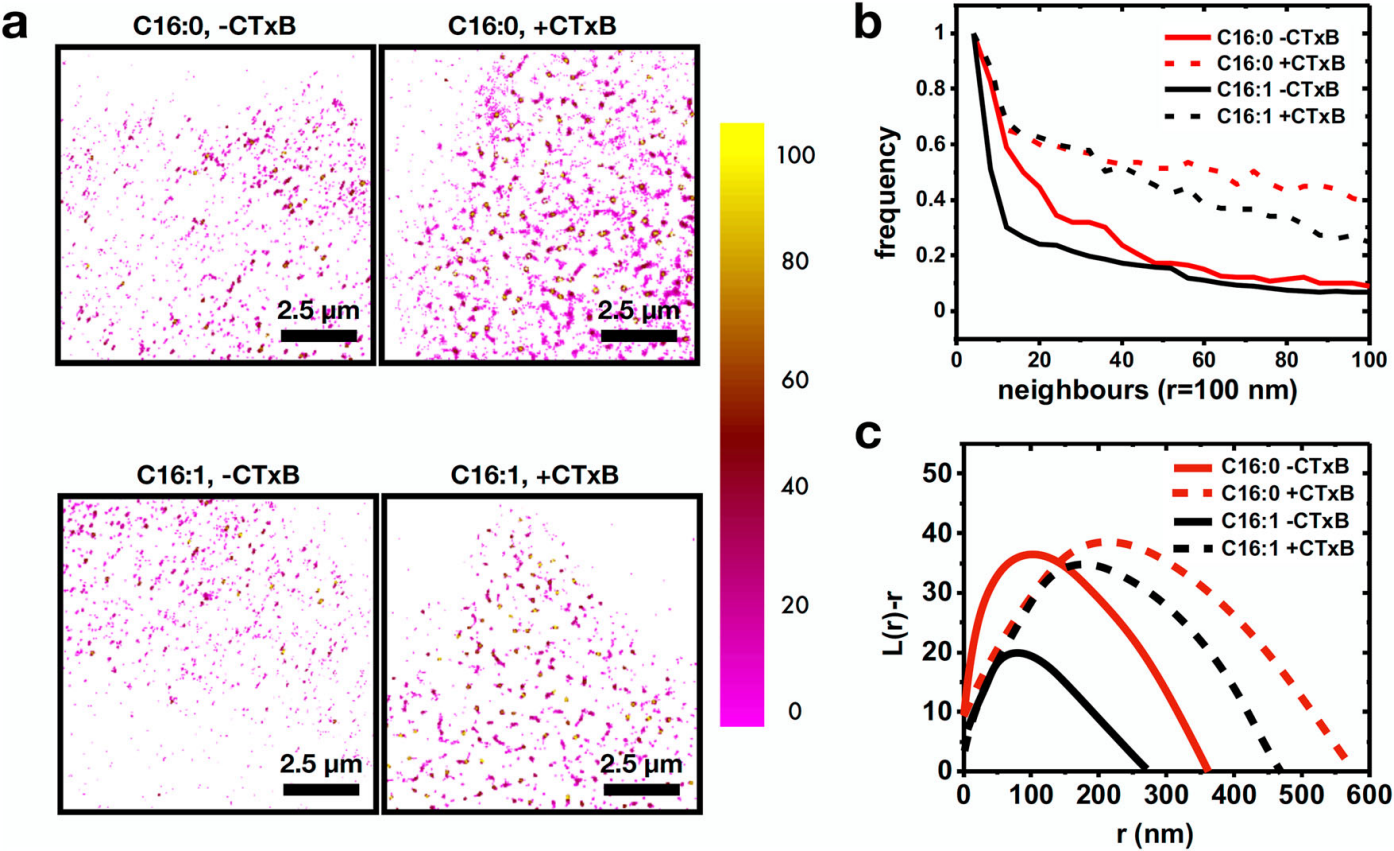
STORM analysis of Alexa488-GM1 clustering. **a** Density maps of Alexa488-GM1 C16:0 (top) and Alexa488-GM1 C16:1 (bottom) in the absence (left) and presence (right) of CTxB. The colormap represents the number of neighbors. **b** Density plots of frequency of occurrence against number of neighbors for Alexa488-GM1 C16:0 (red) and Alexa488-GM1 C16:1 (black) in the presence (dashed) or absence (solid) of CTxB. **c** Ripley’s k-function analysis of distributions of Alexa488-GM1 C16:0 (red) and Alexa488-GM1 C16:1 (black) in the presence (dashed) or absence (solid) of CTxB. For **b** and **c**, *n* = 12 cells from 3 independent experiments.

To further confirm the GM1 clustering induced by CTxB, we performed STochastic Optical Reconstruction Microscopy (STORM) on Alexa488-GM1 C16:0 and C16:1. Alexa488 label on the lipids allowed us to directly measure changes in the spatial organization of the C16:0 or C16:1 GM1 molecules. Alexa488 was stochastically turned on and off to reconstruct super-resolved localizations, as described^50^. To measure the extent of clustering induced by CTxB, we performed density analysis of localizations, as well as Ripley’s K-function analysis. Addition of CTxB to Alexa488-GM1 C16:0 or C16:1-reconstituted cells resulted in both cases in the formation of denser and larger clusters as evidenced in density visualizations of localizations corresponding to number of neighbors (Fig. 4a). Figure 4b represents this graphically. It should be noted that the map depicts the number of neighbors for a given radius. It is therefore representative of the spatial distribution of the localizations, but not of their total number. When treated with CTxB, both C16:0 and C16:1 packed more densely. Concomitantly, Ripley’s K-function analysis applied to stochastic regions of 3 times 3 µm^2^ revealed a shift in the r_max_ values from ~90 and 115 nm for C16:1 and C16:0, respectively, to 190 nm and 225 nm upon CTxB binding (Fig. 4c).

The anisotropy, density, and cluster size changes observed upon CTxB binding strongly suggest that CTxB brings together multiple C16:0 or C16:1 molecules, with these lipid molecules being confined in their rotational diffusion. Further, multiple CTxB molecules bound to the membrane come together, thereby increasing the cluster size.

### Recruitment of GPI-anchored proteins to CTxB-induced domains

**Fig. 5:**
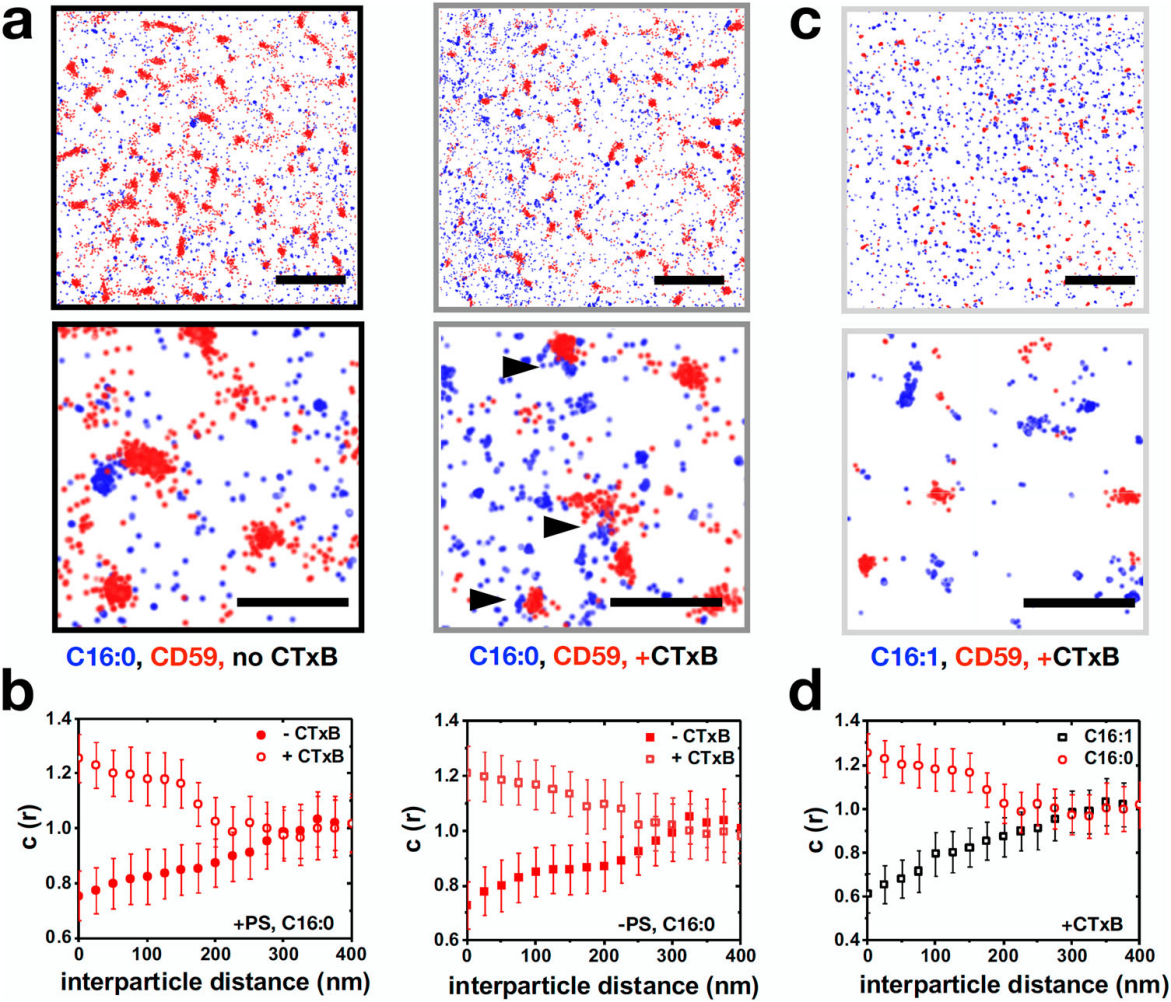
Effect of CTxB on co-clustering of GM1 species with the GPI-anchored protein CD59. **a** Single molecule localization microscopy of mCherry-CD59 and Alexa488-GM1 C16:0 in the presence or absence of CTxB for PS-sufficient cells. **b** Cross-correlation functions, C(r), of mCherry-CD59 and Alexa488-GM1 C16:0 in PS-sufficient (left) or PS-deficient (right) cells, with (open symbols) or without CTxB (solid symbols). **c** mCherry-CD59 and Alexa488-GM1 C16:1 do not co-cluster upon CTxB binding. **d** Cross-correlation functions, C(r), of mCherry-CD59 and Alexa488-GM1 C16:1 (black squares) and C16:0 (red circles) in the presence of CTxB. Scale bars in **a** and **c**: top 1.5 μm, bottom 500 nm. Error bars in **b** and **d** represent SEM between the cells. *n* = 9 cells from 3 independent experiments for **b** and **d**.

The clustering of GPI-anchored proteins is affected by PS^14^, and gangliosides interact with the GPI-anchored receptor CD59^37^, which is likely to be internalized by the GL-Lect mechanism^51^. We therefore investigated how GPI-anchored proteins and CTxB integrate in their ability to co-cluster at the plasma membrane, and if they depend on lipid chain saturation and acto-myosin cortex. Using single molecule localization microscopy, we performed multi-color super-resolution imaging of mCherry-labeled CD59 with Alexa488-labeled GM1 C16:0 or C16:1. We found that mCherry-CD59 clusters did not codistribute with Alexa488-GM1 C16:0 (Fig. 5a, left images). A previous study demonstrated that GM1 molecules transiently colocalized with CD59 with durations of less than 100 ms^37^. This transient nature of colocalization can explain why in our super-resolution imaging-based approach on fixed samples we do not detect their co-distribution. In contrast, upon CTxB addition to C16:0 incorporated cells, mCherry-CD59 and Alexa488-GM1 C16:0 co-distributed (Fig. 5a, right images). We quantified the co-partitioning of CD59 and C16:0 or C16:1 using cross-correlation of point localizations in two different channels that allows analysis of protein co-distributions^52^. We calculated cross-correlation function C(r), as previously described, that measures the relative probability of finding localization pairs separated by distance r as opposed to random distributions. We found that CTxB induced co-distribution for C16:0 and CD59 (Fig. 5b). This co-distribution was not dependent on PS (Fig. 5b), suggesting that CTxB organizes membrane domains by over-riding other clustering mechanisms. Interestingly, Alexa488-GM1 C16:1 did not codistribute with CD59 in the presence of CTxB (Fig. 5c, d). Based on aaMD simulation, this may be explained by the in silico finding that the acyl chain of uGM1 became more disordered upon CTxB binding than that of sGM1 (Supplementary Fig. 7). This increased disorder of the C16:1 acyl chain may exclude CD59 molecules from association with corresponding CTxB domains. The sphingoid base did not experience this disordering (Supplementary Fig. 7).

## Discussion

**Fig. 6:**
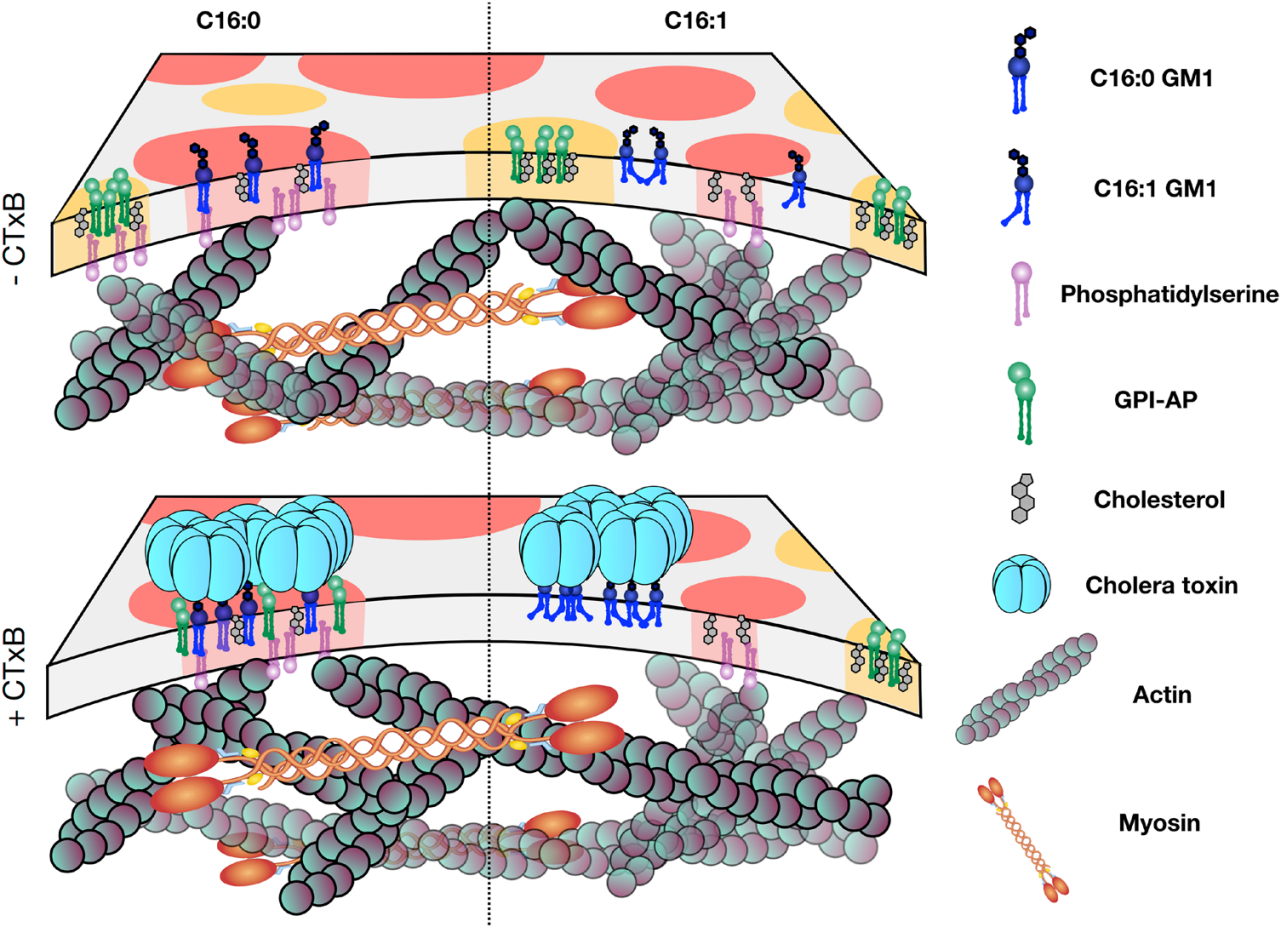
Schematic model of GM1 C16:0 and C16:1 clustering in biological membranes, and changes upon CTxB binding. In the absence of CTxB (top), GM1 C16:0 is found in clusters amenable to active organization dependent on PS and actin (left), while GM1 C16:1 in not clustered (right). Upon CTxB binding (bottom), clustering of underlying lipids occurs for both GM1 C16:0 (left) and GM1 C16:1 (right), independently of actin. Co-clustering with GPI-AP is limited to GM1 C16:0.

GSLs are essential for life by controlling many cellular processes, including endocytosis^53–55^. These functions are thought to depend at least in part upon GSL assembly into raft nanodomains with *trans*-bilayer coupling to the acto-myosin cortex. Our results provide evidence in support of that model. The most striking finding is that the spontaneous formation of GM1 nanoclusters depended critically on the acyl chain structure of the GSL. Only GM1 ceramides containing fully saturated C16:0 acyl chains enabled their spontaneous assembly into nanoclusters, while GM1 with ceramide containing C16:1 acyl chain did not. Membrane cholesterol and PS content, and association with the active actin cortex were also required. We interpret these findings according to a model in which cortical acto-myosin dynamics lead to the molecular focusing of PS molecules which, by *trans*-bilayer coupling, drive the cholesterol-dependent nanocluster formation of saturated GM1 C16:0 molecules in the outer membrane leaflet (Fig. 6, upper schematics, left). We hypothesize that ceramides with C16:1 acyl chains cannot pack against cholesterol in the same way, thus interfering with nanodomain formation (Fig. 6, upper schematics, right).

According to this model, nanoclusters of saturated GM1 C16:0 share several key features with nanoclusters of GPI-APs^14^. Despite this, however, GM1 C16:0 and GPI-AP nanoclusters were segregated under steady state conditions. They colocalized only when GM1 was scaffolded together into larger structures by binding to the extracellular lectin CTxB (Fig. 6, lower schematics). Thus, raft nanodomains of different structures co-exist without necessarily fusing together. Again, most remarkably, even when scaffolded by CTxB into nanodomains, the structure of the ceramide acted decisively to enable co-assembly with GPI-anchored proteins; colocalization with GPI-AP occurred only for the nanodomains containing GM1 ceramides with C16:0 acyl chains (Fig. 6, lower schematics, left). Nanodomains of GM1 C16:1, forced to form by binding CTxB, did not fuse with nanodomains containing GPI-AP (Fig. 6, lower schematics, right). These CTxB-induced nanodomains were also smaller, implying that the GM1 C16:1 *cis*-double bond interfered with the ability of these nanodomains to coalesce with other nanodomains, thereby explaining the lack of colocalization with GPI-AP. We also note that CTxB-induced nanodomains of the GM1 C16:0 species assembled independently of PS. Thus, the strong scaffolding induced by binding CTxB appears to replace the function of *trans*-bilayer coupling to the acto-myosin cortex. It thereby appears that CTxB binding to GM1 lipids strongly influences the structure of the plasma membrane by building domains. Their composition depends on the tail saturation of the GM1, which determines whether other components can co-partition or not. We have shown that CD59 is excluded in the case of C16:1, but co-partitions into CTxB-induced domains for C16:0.

In these studies, we used GM1 with ceramides containing either C16:0 or C16:1 acyl chains. These behave similarly to GM1 species containing C18:0 or C18:1 acyl chains, as evidenced by endosome sorting^29^, and the phase behavior in giant plasma membrane vesicles (Schmieder and Lencer, unpublished). This pair of C16:0 and C16:1 GM1 species was used as they behaved much better for reconstitution compared to GM1 with longer acyl chains, even if they are of low abundance in nature. Of note, other sphingolipids of high abundance in native cell membranes (such as sphingomyelin) have ceramides containing C16:0 and C16:1 acyl chains^56^.

We hypothesize that nanoclusters of GM1 C16:0 lipids may act as nucleation sites at which cholera toxin molecules can efficiently start the membrane binding process. In fact, each of the five binding sites on CTxB has only µM affinity for the carbohydrate part of GM1^57–60^. Efficient membrane association therefore requires multiple bond interactions, which are expected to be favored when several GM1 molecules are in close proximity, such as in nanoclusters. Such binding may also coalesce other GM1-CTx nanoclusters (or include GM1 of other species) to drive the nucleation of endocytic uptake sites^21^. For this, the toxin imposes mechanical effects onto the membrane, some of which have been pioneered for the related Shiga toxin: curvature generation, lipid compression^20,21,24,25,28,61^, and membrane-mediated clustering (^26,62^, and recently^61^ for CTxB). It is likely that a similar generic mechanism may operate for lectins from other glycosphingolipid-binding pathogens like polyomaviruses^16^, and for cellular galectins that upon oligomerization also bind glycosphingolipids in relation to endocytic uptake into cells^23,63^. While it is acknowledged that a plethora of phenomena influence the membrane bilayer, our study emphasizes that both lipid clustering and tail structure set the composition of domains, which is likely exploited for tuning interactions in membrane biological processes.

The general phenomenology of CTxB driving the formation of large clusters as specific functional lipid platforms also hints at richer mechanisms that can override factors that actively or passively maintain the heterogeneous organization of the plasma membrane against temperature or compositional fluctuations. For example, the model for the construction of endocytic sites by lectin binding to GSLs may not be limited to toxins. The cellular galectin-3 has also been shown to drive narrow membrane bending and the construction of tubular endocytic pits in interaction with gangliosides^23^. Furthermore, galectins have been shown to drive the cellular uptake of the GPI-anchored protein CD59^51^, which we have found here to be co-clustered with GM1 upon binding of the latter by CTxB. The possibility thereby arises that galectin-3 (and other galectins) could have a similar effect on the construction of membrane nano-environments in which cargo glycoproteins and GSLs are co-clustered in relation to endocytic uptake into cells.

## Methods

### Cell culture and GSL incorporation into cells

The ethanolamine auxotroph PSA-3 cell line, which lacks PS synthase 1 enzyme was a kind gift from Tomohiko Taguchi (University of Tokyo, Japan). HeLa cells were obtained from American Tissue Culture Collection (ATCC), and GM95 cells from RIKEN Cell Bank, Saitama, Japan. GM95 cells and Hela cells were cultivated in Dulbecco’s modified eagle medium (DMEM) supplemented with 10% fetal bovine serum (FCS), 1 mM sodium pyruvate, 2 mM glutamine, and 1% penicillin/streptomycin (DMEM/FCS). For GSL incorporation a complex of synthetic C16:0 or C16:1 lipid with defatted bovine serum albumin (BSA) was prepared in serum-free DMEM supplemented with 15 mM Hepes (pH 7.2) at a molar ratio of 1:1. Cells were seeded on coverslips and were incubated with the lipid/BSA solution for 2 h at 37 °C. Cells were washed once with DMEM/FCS and once with PBS and were detached with accutase. Cells were collected by centrifugation, resuspended in DMEM/FCS and replated in fibronectin-coated glass-bottom dishes (Mattek) for at least 45 min. For imaging cells were placed in PBS supplemented with 1 mM CaCl_2_, 1 mM MgCl_2_, and 5 mM glucose. For GPMV generation, CaCo BBE were seeded two days prior to the assay on 12-well plates to reach 70% confluency. Native or fluorescently labeled GM1 species were added as described above. A liquid-disordered phase marker (*FAST*DiL™, 0.5 mg/ml, 1:1000 in PBS) was added for 10 min. Where indicated, fluorescently labeled CTxB was added at 5 nM for 15 min. Phase marker was washed off three times using PBS, then cells were equilibrated by washing twice in GPMV buffer (50 mM Tris, pH 8, 150 mM NaCl, 50 mM CaCl_2_).

### GPMV preparation

GPMVs generation was achieved by incubating cells for 3 h at 37 ˚C with 2 mM DTT and 0.07% PFA in GPMV buffer. Supernatant containing GPMV vesicles were harvested and sedimented by centrifugation for 5 min at 500 × *g*. Vesicles were chilled to 10 °C and imaged. For quantifying partitioning of lipid markers in blebs, regions of interest were drawn encapsulating the vesicle boundary and intensities were measured. L_d_ phase was determined by the phase marker, and % L_o_ partitioning coefficient was obtained using the following formula: % L_o_ = I_*Lo*_/(I_*lo*_ + I_*ld*_). A % L_o_ > 0.5 indicates ordered phase preference. For anisotropy studies, blebs were induced by treatment of cells for 30 min with cytochalasin D at 5 µM.

### PSA-3 cells

PSA-3 cells (CHO cells deficient in phosphatidylserine synthesis^47^) were maintained in Ham’s F12 medium supplemented with 10 µM ethanolamine as precursor for PSA-3 cells to synthesize phosphatidylserine. For PS depletion, the PSA-3 cells were grown in the absence of ethanolamine in dialyzed serum for 48 h.

### Treatment of cells with CTxB and quantification of CTxB-surface binding

For quantification of CTxB-surface binding cells were incubated with 50 µg/ml CTxB-AlexaFluor647 for 30 min at 4 °C, washed, fixed with 0.2% glutaraldehyde and 4% PFA in cytoskeletal buffer (10 mM MES, 150 mM NaCl, 5 mM EDTA, 5 mM glucose, and 5 mM MgCl_2_) and mounted in Mowiol. Samples were imaged using a Leica SP8 confocal microscope equipped with a ×63 (N.A. 1.3) objective and appropriate filter sets. Regions of interest were defined by drawing the contours of cells with the free-hand selection tool in ImageJ. Background was defined on cell-free regions on the same coverslips. Mean fluorescence intensities of cells were calculated with the measure tool in ImageJ, and background subtracted.

For fluorescence anisotropy imaging of toxin-bound, fluorescently labeled lipids cells were incubated with 25 µg/ml unlabeled CTxB at 37 °C. The toxin was present during imaging.

### Fluorescence anisotropy imaging and image analysis

Steady state fluorescence anisotropy measurements were essentially carried out as described previously^38^. Briefly, images were acquired using a custom designed anisotropy setup based on a spinning disk confocal setup (Andor revolution, Andor, Belfast, Northern Ireland). Images were acquired using ANDOR EMCCD cameras with a CSU-22 pinhole array disk coupled to a ×100 1.4 NA objective. Imaging was carried out at 37 °C. Image analysis was done in ImageJ. A G-factor correction was performed by measuring the background intensities for the two cameras using fluorescent rhodamine in buffer at 50 mM. For quantification of mean fluorescence anisotropy for plasma membrane regions, mean parallel and perpendicular emission intensities were quantified for regions of interest that were selected based on flat surfaces along the coverslip (Fig. 1c, Supplementary Fig. 4a). Total intensity and anisotropy were calculated in a custom Python code using the following equations. Total intensity = I_pa_ + 2(*g*)I_pe_. Anisotropy: (I_pa_ − (*g*)I_pe_)/(I_pa_ + 2 (*g*)I_pe_). Mean and standard deviations for total intensity and anisotropy were calculated for regions of interest as follows: For each pixel, the total intensity and anisotropy were extracted. These values were sorted in increasing order with corresponding anisotropy values and binned. For each bin, the average intensity and the corresponding average anisotropy was calculated and plotted (Supplementary Fig. 4b). ROIs across multiple cells were pooled together to calculate the mean and standard deviation for a particular average intensity within the bin. Color coded images displaying anisotropy values were prepared using a custom Python code.

### Super-resolution microscopy

Super-resolution microscopy was performed using an Oxford Nanoimager. Cells were fixed after incorporating synthetic Alexa488-tagged GM1 C16:1 or C16:0, with or without addition of CTxB using 0.2% glutaraldehyde and 4% PFA in cytoskeletal buffer (10 mM MES, 150 mM NaCl, 5 mM EDTA, 5 mM glucose and 5 mM MgCl_2_) for 15 min at room temperature^64,65^. The cells were washed gently three times with PBS. For STORM, coverslips were mounted on an Atto Chamber (ThermoFisher) and submerged in 50 mM TRIS, 10 mM NaCl (at pH 8), GLOX (0.5 mg/mL glucose oxidase, 40 μg/mL catalase, 10% glucose) with 100 mM mercaptoethanol. Acquired movies were exported as tiffs, GPU based SMLocalizer was used to reconstruct the images, and localizations were exported to text files to be analysed or visualized. Visualization was performed using ViSP. Density analysis were performed using ViSP with radius set to 100 nm^66^. For Ripley’s K function^67^, the Ripley’s K-cabinet in LocAlization Microscopy Analyser (LAMA)^68^ was used for chosen regions of interest of 3 by 3 µm^2^ of area. Cross-correlations between clusters were performed using a previously published MATLAB code. Extracted data were plotted using ORIGINPRO.

### Fluorescent lipid synthesis

GM1 containing either C16:0 or C16:1 fatty acid was provided by Prof. S. Sonnino. The linker peptide was custom synthesized by New England Peptide, Inc., with the following amino acid sequence: propargylglycine-k(ε-biotinoyl)(ds)g(dy)g(dr)g(ds)g-(kaoa)-amine. Gangliosides were labeled on the oligosaccharide headgroup according to^69^. In short, ~1300 nmoles of respective GM1 was oxidized with 13 mmoles sodium periodate in 100 mM sodium acetate pH 5.5, 150 mM NaCl for 30 min on ice. The reaction was quenched by addition of glycerol to 5% and purified over Elut SepPak C18 cartridge (Agilent, MA). The oxidized product was reacted with 2700 nmoles of aminooxy-containing peptide in 2 mL PBS pH 6.9, 10% DMF and 10 mM aniline for 12 h at room temperature. The precipitate was separated from the solution by centrifugation and resuspended in 100 uL 50% isopropanol/water. The oxime bond was reduced with 4.8 mmoles of sodium cyanoborohydride for 3 h. Lipid-peptide conjugates were purified by semi-preparative HPLC, and masses were confirmed by either MALDI-TOF (AB Voyager), or ESI LC-MS (Agilent, MA). 320 uM peptide-lipid molecules were reacted with equimolar concentrations of Alexa Fluor 488–azide in 50 mM Tris-Cl, 5 mM copper (II) sulfate, 100 mM sodium ascorbate, 37 mM (Tris[(1-benzyl-1H-1,2,3-triazol-4-yl)methyl]amine, TBTA in DMSO/t-butanol 1:4) 1 mM (Tris(2-carboxyethyl) phosphine hydrochloride, TCEP—Sigma) for 12 h at room temperature. Products were purified by semi-preparative HPLC and confirmed by mass spectrometry.

### Incorporation of fluorescent GM analogues in live cells

To study GSL organization in the plasma membrane of living cells, GM95 cells were supplemented with fluorescently labelled versions of GM1. Synthetic GM1 was added exogenously by incubating the cells with a GM1/BSA complex. This protocol was shown previously to result in a functional incorporation of synthetic lipid analogues into the plasma membrane with GM1 showing expected diffusion characteristics and enabling binding and endocytosis of CTxB^29^. GM95 cells do not express endogenous GSLs due to a mutation in the glucosylceramide synthase gene, whose product catalyzes the first step in synthesis of glucosylceramide-based GSLs^70^. The synthetic analogues are hence the only GSLs present, which allows comparing the clustering behavior of defined GM1 species. Four GM1 versions were used throughout the study, two lipid species labeled with AlexaFluor488 (Alexa488-GM1 C16:0 and Alexa488-GM1 C16:1) and two nonfluorescent versions (GM1 C16:0 and GM1 C16:1).

### All-atom molecular dynamics simulations

We performed aaMD simulations of asymmetric bilayers containing around 4% GM1 lipids in upper monolayer. We first explored the behavior of GM1 in a simple bilayer containing 96% DOPC lipids, in presence or absence of CTxB. Two types of GM1 species were simulated: GM1 with a fully saturated acyl chain (C16:0; termed sGM1), and unsaturated GM1 with a double bond at carbon 9 of its acyl chain (C16:1; termed uGM1). In all systems, GM1 lipids were only in the upper monolayers (for more details see Supplementary Table 1). Then, we investigated the behavior of both GM1 lipid types in a smaller but more complex asymmetric bilayer containing POPC, CHOL, and sphingomyelin (SSM) in the upper monolayer, and POPE, POPS, and CHOL in the lower monolayer. Additionally, we performed two control simulations in which no cholesterol molecules were present (for more details see Supplementary Table 2). To build asymmetric bilayers, we first preformed simulations of smaller symmetric bilayers for three different GM1 concentrations (6, 12, or 18 mol%), and both GM1 types. Using these results, we obtained area per lipid values for GM1 and DOPC, which were then used to match (as much as possible) the total area of different monolayers of the asymmetric bilayers. We do note that this does not guarantee a tensionless bilayer; however, this is as good as we can do for asymmetric bilayers at atomistic resolutions^71,72^. The simulations were performed using GROMACS^73,74^ and the CHARMM36 force field^75,76^. The systems were solvated using the TIP3P water model^77^. Since GM1 (1 negative charge), POPS (1 negative charge), and CTxB (2 positive charges per monomer) are charged molecules, K+ ions were added to neutralize the system. Electrostatic interactions were treated with particle-mesh Ewald (PME), with a short-range cutoff at 1.2 nm, and van der Waals interactions were switched off between 1.0 and 1.2 nm. The system temperature was kept constant at 37 °C using Nose-Hoover temperature coupling^78,79^. Bonds containing hydrogen atoms were constrained using the LINCS algorithm^80^. Parrinello-Rahman barostat pressure coupling^81^ was applied to all systems after equilibrating them with the Berendsen pressure coupling^82^. Finally, the leapfrog integrator was used with a time step of 2 fs.

### Reporting summary

Further information on research design is available in the Nature Research Reporting Summary linked to this article.

## Supplementary information

Supplementary Information

Reporting Summary

## Data Availability

The data supporting the findings of this study are available upon reasonable request from the corresponding author. Source data are provided with this paper.

## Source data

Source Data

## Code availability

Custom codes for anisotropy analysis are available at https://github.com/zeroth/Anisotropy.
