# Supplementary material for: Ceramide structure dictates glycosphingolipid nanodomain assembly and function
Source: Nat Commun. 2021 Jun 16;12:3675. doi: 10.1038/s41467-021-23961-9 (PMC8209009; doi:10.1038/s41467-021-23961-9)
Supplement: Supplementary file 1 — Supplementary Information [file 41467_2021_23961_MOESM1_ESM.pdf]

Supplementary Information

Ceramide structure dictates glycosphingolipid nanodomain assembly and function

Senthil Arumugam*,[1,2,3,4], Stefanie Schmieder*,[5], Weria Pezheshkian*,[6], Ulrike Becken[1], Christian Wunder[1], Dan Chinnapen[5], John Hjort Ipsen[7], Anne K. Kenworthy[8], Wayne Lencer[5,9,10]#, Satyajit Mayor[2]#@, Ludger Johannes[1]#@.

[1] Institut Curie, PSL Research University, U1143 INSERM, UMR3666 CNRS, Cellular and Chemical Biology unit, 26 rue d'Ulm 75248 Paris Cedex 05, France.
[2] National Centre for Biological Sciences (NCBS), Bangalore, India
[3] Monash Biomedicine Discovery Institute, Faculty of Medicine, Nursing and Health Sciences, Monash University, Clayton/Melbourne, VIC 3800, Australia
[4] European Molecular Biological Laboratory Australia (EMBL Australia), Monash University, Clayton/ Melbourne, VIC 3800, Australia
[5] Division of Gastroenterology, Boston Children's Hospital, Boston MA 02115
[6] Groningen Biomolecular Sciences and Biotechnology Institute and Zernike Institute for Advanced Materials, University of Groningen, Groningen, Netherlands.
[7] MEMPHYS/PhyLife, Department of Physics, Chemistry and Pharmacy, University of Southern Denmark, Campusvej 55, 5230 Odense M, Denmark
[8] Center for Membrane and Cell Physiology, University of Virginia, Charlottesville, VA USA
[9] Harvard Medical School, Boston MA 02115
[10] Harvard Digestive Diseases Center, Boston, MA 02115

* These authors contributed equally.
# These authors jointly supervised this work.
@ Correspondence to: mayor@ncbs.res.in and ludger.johannes@curie.fr

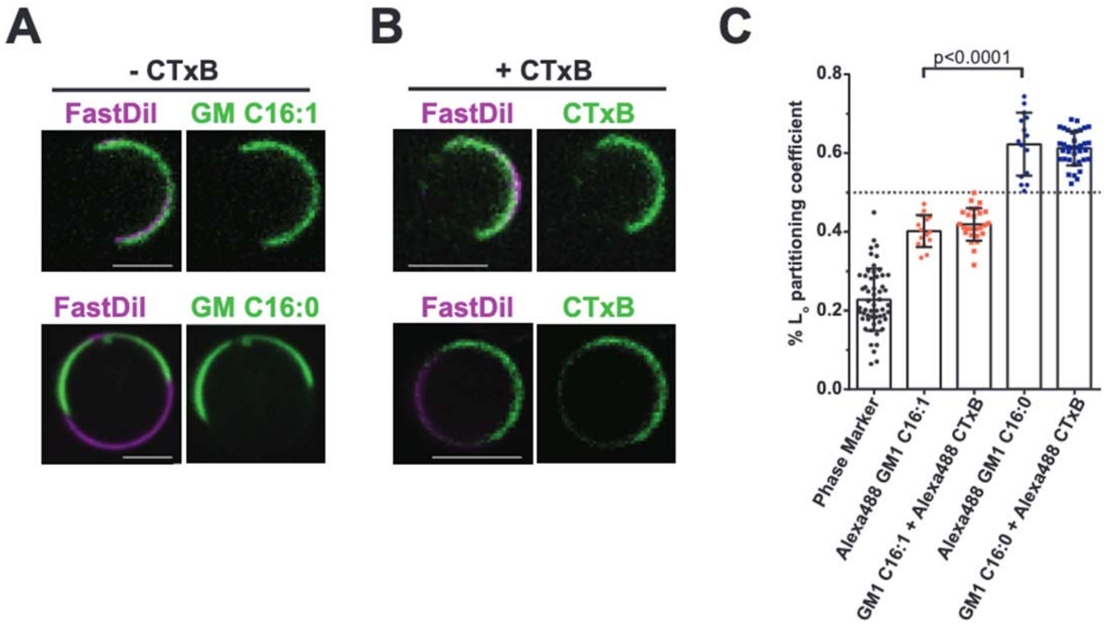

Supplementary Figure 1: Phase behavior of the different GM1 species in giant plasma membrane vesicles (GPMVs) derived from CaCo BBE cells with and without 5 nM of CTxB. (**A**) Representative images of GPMVs in the absence of CTxB (-CTxB). Top panel: GPMV- incorporated with peptide-labelled Alexa488 GM1 C16:1 (green) and Fast DiI (Magenta), in the merged image to the left. Bottom panel: GPMV-incorporated with peptide-labelled Alexa488 GM1 C16:0 (green) and Fast DiI (magenta) in the merged image to the left. (**B**) Representative images of GPMVs in the presence of Alexa488 labeled CTxB (+CTxB). Top panel: GPMV-incorporated with unlabeled GM1 C16:1 in the presence of CTxB-A488 (green), and Fast DiI (magenta) in the merged image to the left. Bottom panel: GPMV-incorporated unlabeled GM1 C16:0 in the presence of CTxB-A488 (green), and Fast DiI (magenta) in the merged image to the left. Scale bars = 5 µm. (**C**) Partitioning coefficients for the indicated conditions strongly suggest that labeled and unlabeled GM1 species have the same phase behavior. Bars represent means ± SD, for n=5 independent experiments. Per experiment, 3-10 GPMVs were imaged and quantified. One-way ANOVA and Tukey's multiple comparisons were performed.

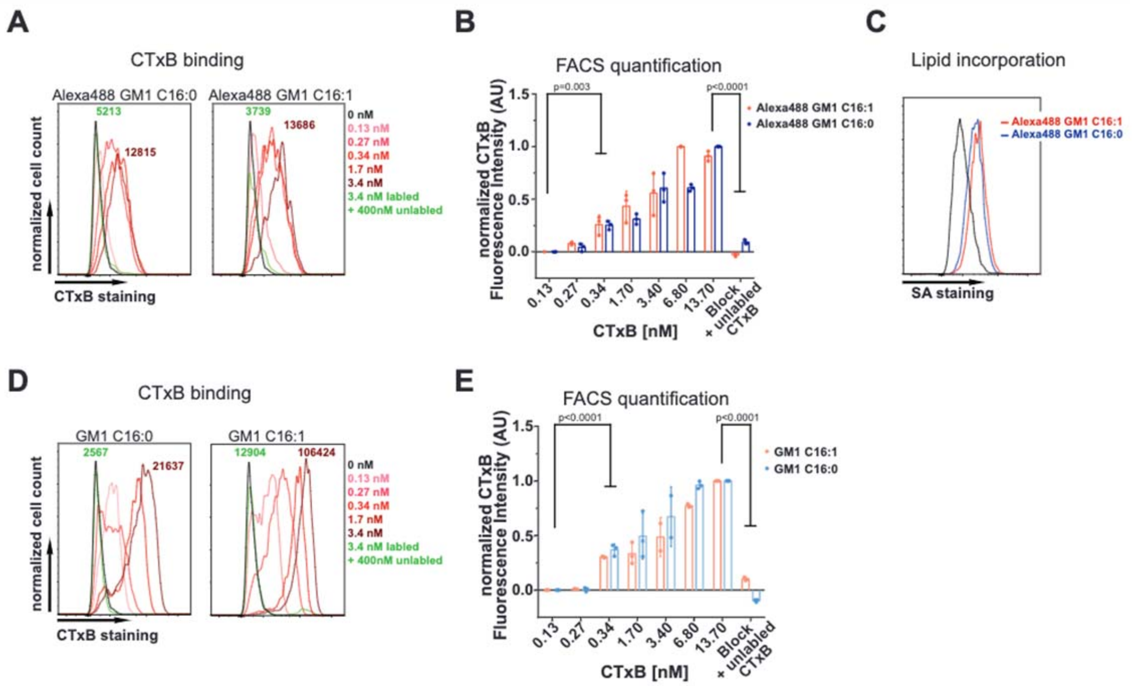

Supplementary Figure 2: Nanomolar binding of CTxB to synthetic and native GM1. (**A**) CaCo BBE cells were loaded for 10 min at 37 °C with 0.25 uM of peptide-labeled Alexa488 GM1 C16:1, 2 µM of peptide-labeled Alexa488 GM1 C16:0. Lipids were washed off, cells were transferred for 10 min to 4 °C, and then incubated for 15 min at 4 °C with different concentrations (0, 0.13, 0.27, 0.34, 1.7, 3.4 nM) of CTxB-Alexa647 in PBS + 1 % BSA. A control (block) containing 3.4 nM fluorescent CTxB was competed with 400 nM unlabeled CTxB (light green Alexa488 GM1 C16:1, dark green Alexa488 GM1 C16:0). Cells were washed twice before FACS sorting. 5,000 cells were sorted per treatment. Means for both block and 3.4 nM fluorescent CTxB are indicated in graph. (**B**) Quantification of normalized FACS data from (A). Alexa488 GM1 C16:1 is shown in red, and Alexa488 GM1 C16:0 in blue. Bars represent means ± SD for n=3 independent experiments. Two-way ANOVA with Tukey's multiple comparison was performed. (**C**) Lipid incorporation. Equal loading between Alexa488 GM1 C16:1 in red and Alexa488 GM1 C16:0 in blue was verified using fluorescently labeled streptavidin (SA). (**D**) CaCo BBE cells were loaded as in (A) with 1 µM of unlabeled GM1 C16:1, or 5 µM of unlabeled GM1 C16:0. CTxB binding was analyzed. 5,000 cells were sorted per condition. (**E**) Quantification of CTxB binding from (D). GM1 C16:1 in red and GM1 C16:0 in blue, the block condition is shown in light green for GM1 C16:1, and dark green for GM1 C16:0. Bars represent means ± SD, for n=3 inpendent experiments. Two-way ANOVA with Tukey's multiple comparison was performed.

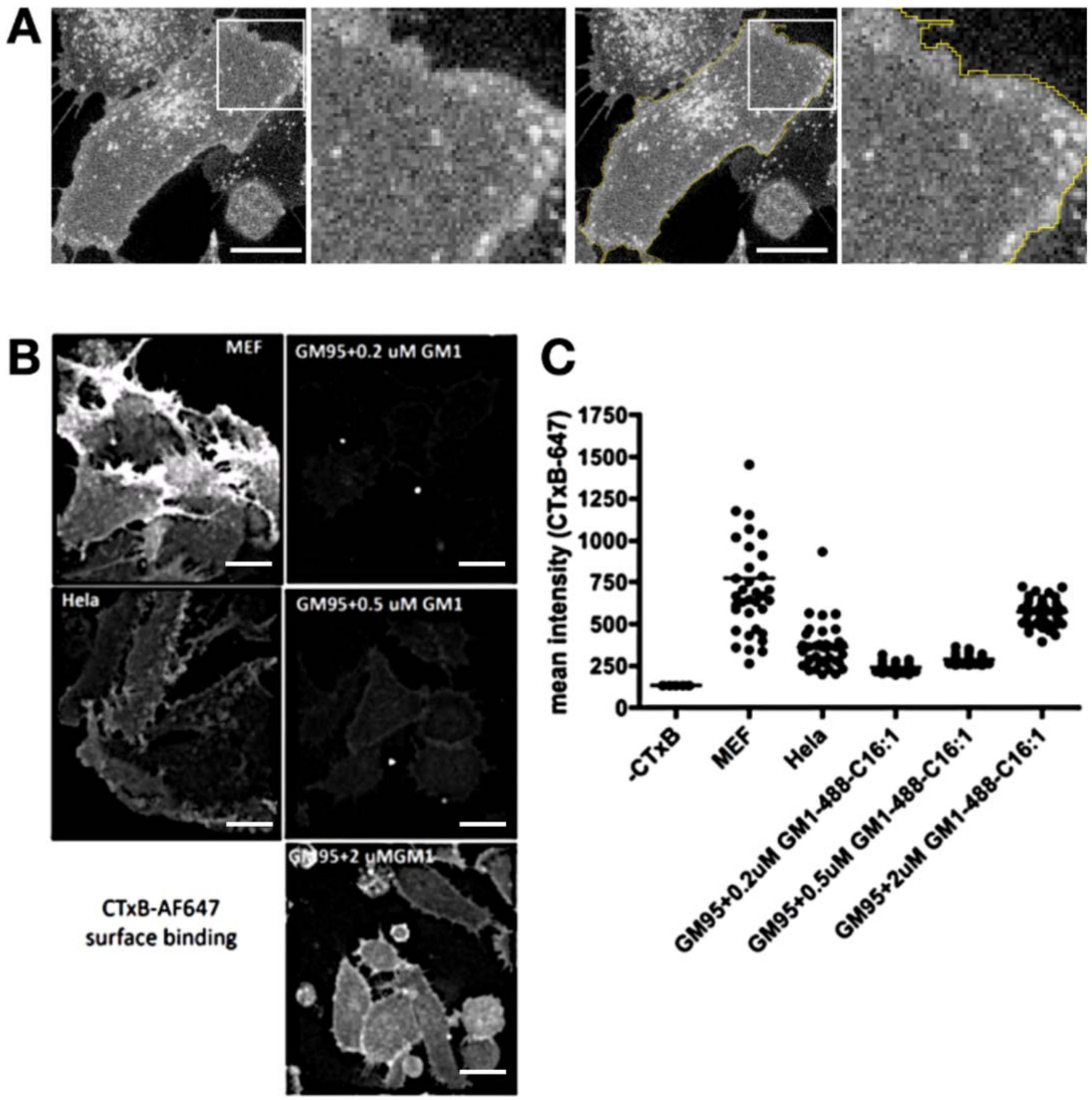

Supplementary Figure 3: Incorporation of GM1 analogues in live cells. (**A**) Example of manual outline of a cell using the freehand selection tool in ImageJ. (**B**) Example images of GM1 incorporated in GM95 cells at different concentrations, as compared to HeLa cells and MEFs[1,2]. Scale Bars: 10 μm. (**C**) Quantification of binding of CTxB to HeLa cells, MEFs, or GM95 cells that were incorporated with fluorescent analogues of GM1.

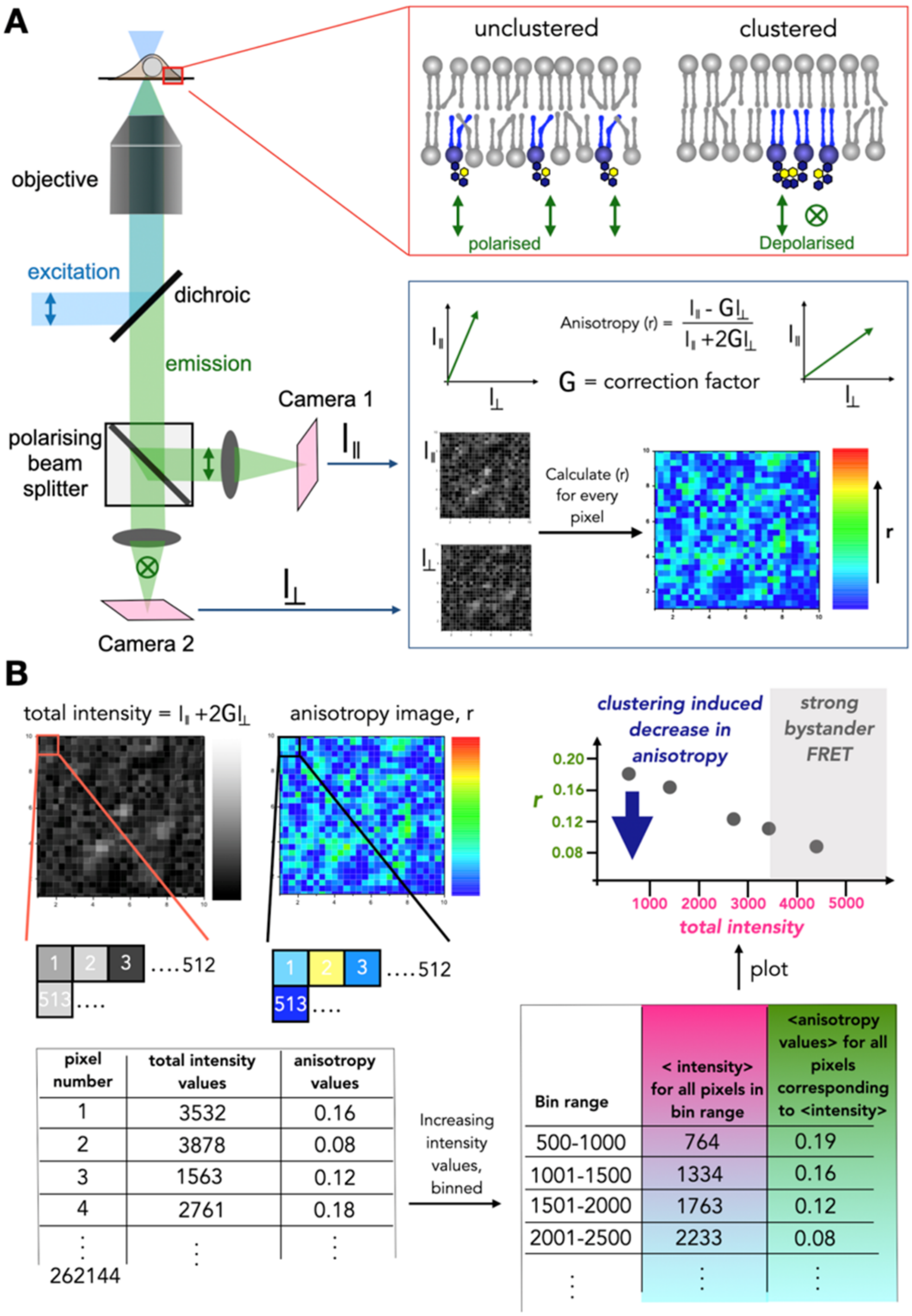

Supplementary Figure 4: Schematic explaining homo-FRET microscopy. (**A**) The key optical layout of homo-FRET microscopy. Polarized light excites fluorophores in the cell sample through an objective, which results in emission. The collected emission carries information on whether or not clusters are present within the resolution limits of the system. This can be retrieved by splitting the emission into perpendicular and parallel polarization using a polarizing beam splitter

and imaging using an EMCCD camera. Anisotropy is calculated using the two images as per the formula, with a correction factor for the grating. This calculation is performed for every pixel resulting in anisotropy images that are color coded. (**B**) Workflow of extracting anisotropy plots from the images. For each pixel within the region of interest, the total intensity and corresponding anisotropy are extracted. An example for 512 X 512 pixels is depicted. This data is then binned according to the intensity ranges, with average intensity values and corresponding anisotropy values for pixels corresponding to each bin calculated. This data can now be plotted as total intensity vs anisotropy. At higher intensities, which represents large number of fluorescently labelled molecules, bystander FRET or random proximity effect can be seen resulting in lower anisotropy values. At lower intensities, anisotropy is sensitive to clustering, displaying lower values when clusters are present, and higher values when clustering is absent. More details can be found elsewhere[3,4,5].

Supplementary Note:

Molecular Dynamics Simulations

Lipid compositions

All-atom molecular dynamics simulation of GM1 in DOPC bilayers in the presence or absence of CTxB.

System	DOPC/sGM1/uGM1/CTxB	DOPC/sGM1/uGM1/CTxB	Solvent	Ion (K^+)	Time
1: Control1	207/18/0/0	223/0/0/0	29564	8	1µs
2: Control2	207/0/18/0	223/0/0/0	29826	8	1µs
3: CTxBS-GM1	207/18/0/1	223/0/0/0	38867	8	1µs
3: CTxBS-GM1	207/0/18/1	223/0/0/0	36860	8	1µs

Supplementary Table 1: Simulated systems. Second column shows upper monolayer content and third column lower monolayer content. sGM1 and uGM1 are GM1 species with ceramide structures containing C16:0 or C16:1 acyl chains, respectively.

All-atom molecular dynamics simulation of GM1 in complex membranes.

Systems	DOPC/SSM/CHOL/sGM1/uGM1	POPE/POPS/CHOL	water	Ion(K^+)	Time
sGM1-CHOL	100/24/60/16/0	85/64/65	18313	80	0.5µs
uGM1-CHOL	100/24/60/0/16	85/64/65	15156	80	0.5µs
sGM1	100/24/0/16/0	84/64/0	18313	80	0.5µs
uGM1	100/24/0//16	84/64/0	15156	80	0.5µs

Supplementary Table 2: Simulated systems. Second column shows upper monolayer content and third column lower monolayer content. sGM1 and uGM1 are GM1 species with ceramide structures containing C16:0 or C16:1 acyl chains, respectively.

Level of the interdigitation

The level of the interdigitation (LID) is calculated as follows. First, number density profiles of the last three atoms of each chain are obtained. Then, integral of the density profile per lipid that has crossed the bilayer center, multiplied by total area of the bilayer, is the LID.

$$\mathrm{LID} = -\frac{A}{N_{lipid}} \int_{\mp\infty}^{0} \rho(z)dz$$

where, −(+) is for the outer (inner) leaflet lipids.

GM1 interdigitation in the absence of CTxB

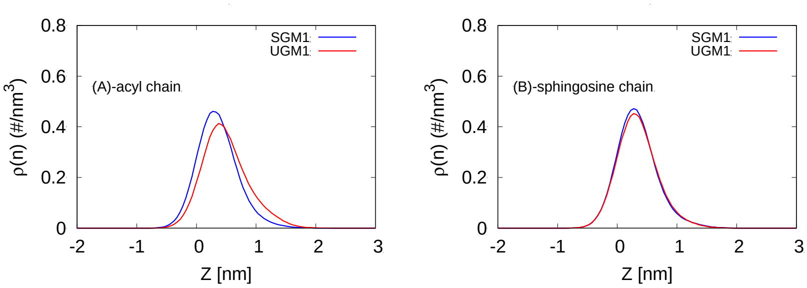

Supplementary Figure 5: Number density profiles for different GM1 chains in GM1/DOPC systems. (**A**) Fully saturated acyl chains interdigitate more into the opposite monolayer. (**B**) Sphingosine chains of both GM1 types interdigitate equally into the opposite monolayer.

Level of interdigitations (particle per lipid)	Acyl chains	Sphingosine chains
uGM1	0.25 ± 0.05	0.45 ± 0.06

sGM1	0.47 ± 0.09	0.43 ± 0.05

Supplementary Table 3: The level of interdigitations (particles per lipid) for different GM1 species in the GM1/DOPC system (the error bar is standard deviation).

Different lipids	sGM1-CHOL	sGM1-no CHOL	uGM1-CHOL	uGM1-no CHOL
GM1	0.17 ± 0.05	0.40 ± 0.04	0.11 ± 0.03	0.39 ± 0.06
SSM	0.44 ± 0.05	0.79 ± 0.09	0.44 ± 0.06	0.91 ± 0.08
POPC	0.43 ± 0.02	0.76 ± 0.02	0.41 ± 0.02	0.82 ± 0.02
POPE	0.94 ± 0.04	1.35 ± 0.02	1.06 ± 0.03	1.38 ± 0.05
POPS	0.77 ± 0.03	1.09 ± 0.05	0.8 ± 0.04	1.14 ± 0.06

Supplementary Table 4: The level of interdigitations (particles per lipid) for different lipids in lipid mixtures containing different GM1 species (see Supplementary Table 2). In all systems, sGM1 interdigitates more than uGM1. Presence of cholesterol (CHOL) reduces interdigitation of all lipids, but increases the difference between sGM1 and uGM1 interdigitation.

System	***Total Area***
sGM1-no CTxB	148.7032 nm^2
sGM1+CTxB	149.2998 nm^2
uGM1-no CTxB	149.8317 nm^2
uGM1+CTxB	149.9582 nm^2

Supplementary Table 5: Calculating area per lipid of GM1. The simulation results show that the total area of an sGM1 system increases by 59.7A^2 upon binding of CTxB, while for the uGM1 system, this increase is only 12.6 A^2. To convert these values to area per GM1 lipid, we assume that the area per lipid of DOPC remains unchanged upon CTxB binding. In this case, the change of the sGM1 area per lipid upon CTxB binding is 3.3 A^2, while for uGM1 it is only 0.7A^2. We do note that assuming that DOPC area per lipid remains unaffected is somewhat approximative. However, other methods for calculating area per lipid such as APL@voro are also associated with high error for this system, as the GM1 concentration is low.

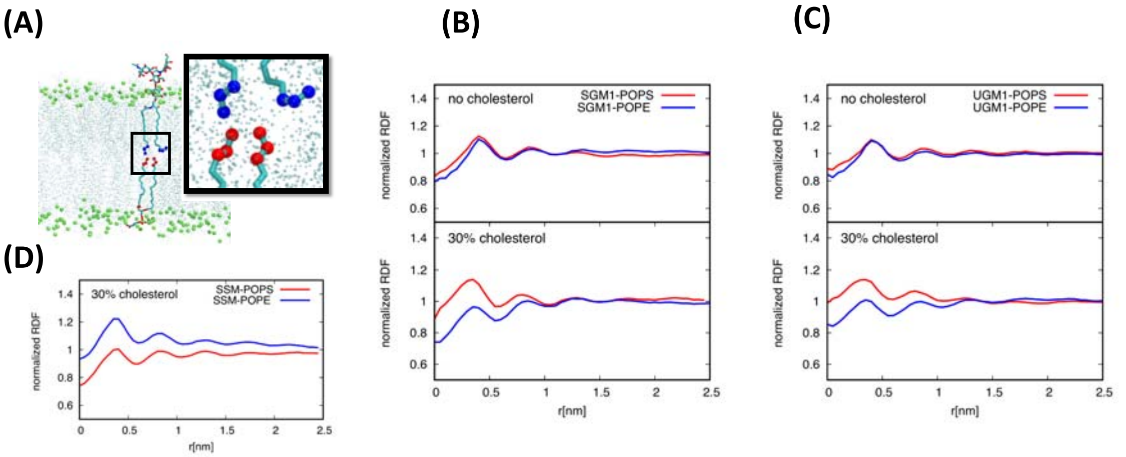

Supplementary Figure 6: **Trans-bilayer registry.** (**A**) Representation of the last three carbon atoms of each chains that are used to obtain the in-plane radial distribution function (RDF). (**B**) RDF of POPS and POPE lipids with respect to sGM1 in the presence (bottom) or the absence of cholesterol (top). (**C**) RDF of POPS and POPE lipids with respect to uGM1 in presence (bottom) or absence of cholesterol (top). (**D**) RDF of POPS and POPE lipids respect to SSM in presence of cholesterol.

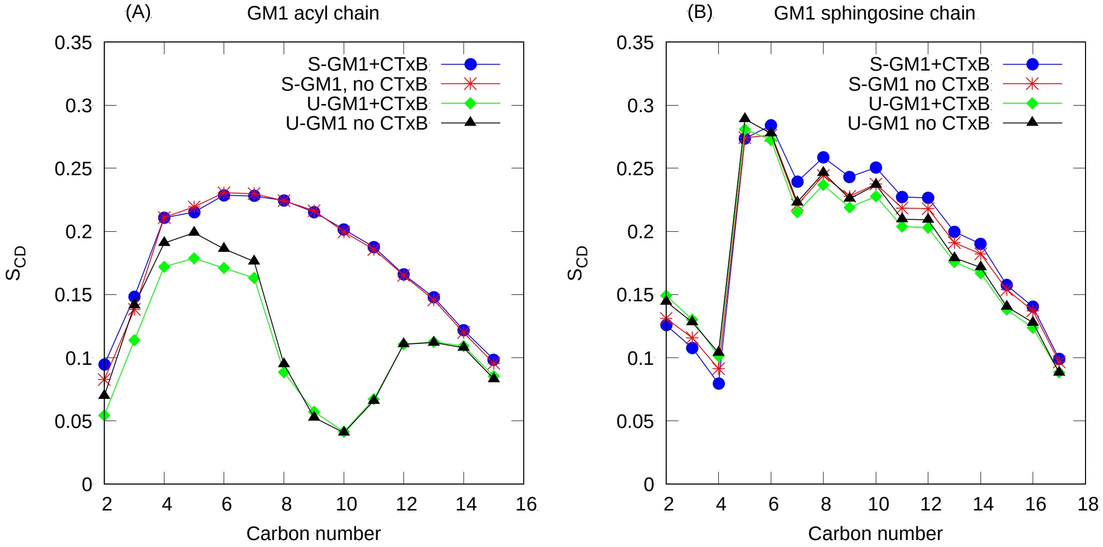

Supplementary Figure 7. Deuterium order parameter. (**A**) Acyl chains and (**B**) sphingosine chains of GM1 lipids in the absence (red and black) or in the presence (blue and green) of CTxB. The results show that the acyl chain of uGM1 becomes more disordered after CTxB binding, while the acyl chain of sGM1 remains unaffected. Note: These results are an average over all the GM1 lipids (18 lipids), which smears the effects of CTxB binding.

Supplementary References:

1. Chinnapen, D.J. *et al.* Lipid sorting by ceramide structure from plasma membrane to ER for the cholera toxin receptor ganglioside GM1. *Dev. Cell* **23**, 573-586 (2012).
2. Ichikawa, S., Nakajo, N., Sakiyama, H. & Hirabayashi, Y. A mouse B16 melanoma mutant deficient in glycolipids. *Proc. Natl. Acad. Sci. USA* **91**, 2703-2707 (1994).
3. Saha *et al.* Homo-FRET imaging highlights the nanoscale organization of cell surface molecules. *Methods in Molecular Biology*, Chapter 9, 151 – 173, (2015).
4. Chan *et al.* HomoFRET Fluorescence Anisotropy Imaging as a tool to study Molecular Self-assembly in Live Cells. *ChemPhysChem*, *12*, 500 – 509, (2011).
5. Ghosh, Saha *et al.* Dynamic Imaging of Homo-FRET in Live cells by Fluorescence Anisotropy Microscopy. *Methods in Enzymology*, Vol 505, 291-327 (2012).
