# Supplementary material for: Ceramide structure dictates glycosphingolipid nanodomain assembly and function
Source: Nat Commun. 2021 Jun 16;12:3675. doi: 10.1038/s41467-021-23961-9 (PMC8209009; doi:10.1038/s41467-021-23961-9)
Supplement: Supplementary file 2 — Reporting Summary [file 41467_2021_23961_MOESM2_ESM.pdf]

nature research

Corresponding author(s): Satyajit Mayor, Ludger Johannes

Last updated by author(s): 15-05-2021

Reporting Summary

Nature Research wishes to improve the reproducibility of the work that we publish. This form provides structure for consistency and transparency in reporting. For further information on Nature Research policies, see our Editorial Policies and the Editorial Policy Checklist.

Statistics

For all statistical analyses, confirm that the following items are present in the figure legend, table legend, main text, or Methods section.

n/a	Confirmed	
☐	☒	The exact sample size (n) for each experimental group/condition, given as a discrete number and unit of measurement
☐	☒	A statement on whether measurements were taken from distinct samples or whether the same sample was measured repeatedly
☐	☒	The statistical test(s) used AND whether they are one- or two-sided<br>*Only common tests should be described solely by name; describe more complex techniques in the Methods section.*
☒	☐	A description of all covariates tested
☒	☐	A description of any assumptions or corrections, such as tests of normality and adjustment for multiple comparisons
☐	☒	A full description of the statistical parameters including central tendency (e.g. means) or other basic estimates (e.g. regression coefficient) AND variation (e.g. standard deviation) or associated estimates of uncertainty (e.g. confidence intervals)
☒	☐	For null hypothesis testing, the test statistic (e.g. F, t, r) with confidence intervals, effect sizes, degrees of freedom and P value noted<br>*Give P values as exact values whenever suitable.*
☒	☐	For Bayesian analysis, information on the choice of priors and Markov chain Monte Carlo settings
☒	☐	For hierarchical and complex designs, identification of the appropriate level for tests and full reporting of outcomes
☒	☐	Estimates of effect sizes (e.g. Cohen's d, Pearson's r), indicating how they were calculated

Our web collection on statistics for biologists contains articles on many of the points above.

Software and code

Policy information about availability of computer code

Data collection	Commercial softwares used: LabView, Oxford Nanoimager, Zeiss Elyra.
Data analysis	OriginLab, Zeiss Elyra, ViSP AND FIJI (Super-resolution), Custom codes for calculating anisotropy with background correction and a range of false colour coding for range (GitHub link in the manuscript).

For manuscripts utilizing custom algorithms or software that are central to the research but not yet described in published literature, software must be made available to editors and reviewers. We strongly encourage code deposition in a community repository (e.g. GitHub). See the Nature Research guidelines for submitting code & software for further information.

Data

Policy information about availability of data

All manuscripts must include a data availability statement. This statement should provide the following information, where applicable:

- Accession codes, unique identifiers, or web links for publicly available datasets
- A list of figures that have associated raw data
- A description of any restrictions on data availability

Provide your data availability statement here.

Field-specific reporting

April 2020

Life sciences study design

All studies must disclose on these points even when the disclosure is negative.

Sample size	No statistical method was used to predetermine sample size. Sample size were chosen based on statistical evaluation of the data post acquisition and strength of effect observed.
Data exclusions	Data exclusions were not pre-established. In the Blebbing experiments, Cells that underwent excessive blebbing and appeared abnormal/ unhealthy were excluded. Including this data did not have an effect on the results, but were excluded to have consistent quality of cells across the measurements.
Replication	All attempts at replication of data contained in this manuscript were successful. Experiments were performed at least three times unless otherwise noted. The crucial experiments where contrasting trends to expected were observed , i.e. increase in anisotropy upon clustering, which now we have described owing to ordering of lipid molecules, were repeated by two independent researchers - SA and UB across a two year period.
Randomization	No method of randomization is applicable here.
Blinding	Experiments are not blinded because the experiments and analysis were performed by the authors, and it was not possible to analyse datasets blindly.

Reporting for specific materials, systems and methods

We require information from authors about some types of materials, experimental systems and methods used in many studies. Here, indicate whether each material, system or method listed is relevant to your study. If you are not sure if a list item applies to your research, read the appropriate section before selecting a response.

Materials & experimental systems

n/a	Involved in the study	
☒	☐	Antibodies
☐	☒	Eukaryotic cell lines
☒	☐	Palaeontology and archaeology
☒	☐	Animals and other organisms
☒	☐	Human research participants
☒	☐	Clinical data
☒	☐	Dual use research of concern

Methods

n/a	Involved in the study	
☒	☐	ChIP-seq
☒	☐	Flow cytometry
☒	☐	MRI-based neuroimaging

Eukaryotic cell lines

Policy information about cell lines

Cell line source(s)	PSA3 cell line Ethanolamine auxotroph which lacks PS synthase 1 enzyme Tomohiko Taguchi (University of Tokyo, Japan). Nishijima, M., Kuge, O., and Akamatsu, Y. (1986). Phosphatidylserine biosyn- thesis in cultured Chinese hamster ovary cells. I. Inhibition of de novo phos- phatidylserine biosynthesis by exogenous phosphatidylserine and its efficient incorporation. J. Biol. Chem. 261, 5784–5789. Lipids at cell surface characterized by Mass spectrometry for these cells are described in Raghupathy et al. Cell, 2015 ; 161(3), 581-594
Authentication	Authenticated
Mycoplasma contamination	All cell lines tested negative for Mycoplasma contamination.
Commonly misidentified lines (See ICLAC register)	NA
